# Characterizing the physical and mental health profile of children, adolescents and adults with autism spectrum disorder in Spain

**DOI:** 10.3389/fpsyt.2023.1088727

**Published:** 2023-03-08

**Authors:** Ruth Vidriales-Fernández, Marta Plaza-Sanz, Cristina Hernández-Layna, María Verde-Cagiao, Guillermo Benito-Ruiz, Fernando Carvajal-Molina

**Affiliations:** ^1^Confederación Autismo España, Madrid, Spain; ^2^Facultad de Ciencias de la Salud, Universidad Isabel I, Burgos, Spain; ^3^Facultad de Psicología, Departamento de Psicología Biológica y de la Salud, Universidad Autónoma de Madrid, Madrid, Spain

**Keywords:** autism spectrum disorder, health conditions, epidemiological factors, mental health, psychopharmacological treatment, Spain

## Abstract

**Introduction:**

Autistic men and women are more likely to experience health issues than the general population, although the available epidemiological studies addressing co-occurrence conditions are limited. This is the first Spanish epidemiologic study addressing the health profile and poor-health exacerbating factors in individuals of all ages with autism spectrum disorder (ASD).

**Methods:**

We analyzed 2,629 registries extracted from Autism Spain’s sociodemographic registry (November 2017–May 2020). A descriptive health data analysis was conducted to assess the prevalence of other conditions associated to ASD in the Spanish population. Nervous system disorders (12.9%), mental health diagnoses (17.8%), and other comorbidities (25.4%) were reported. Men-to-women ratio was 4:1.

**Results:**

Women, elder individuals and those with intellectual disability (ID) were at an increased risk of health comorbidities and psychopharmacological exposure. Women were also more prone to severe intellectual and functional impairment. Nearly all individuals had difficulties in their adaptative functioning, especially those with ID (50% of the population). Almost half of the sample received psychopharmacological treatments starting from infancy and early childhood, mostly antipsychotics and anticonvulsants.

**Discussion:**

This study represents an important first approach to the health status of autistic people in Spain and can contribute to the development of public policies and innovative health strategies.

## 1. Introduction

Autism spectrum disorders (ASD) encompass several neurodevelopmental chronic conditions with early childhood onset that may be, or not, accompanied with an intellectual disability (ID) or language impairment (1, 2). The prevalence of ASD is currently estimated at 1%, although that estimation is variable, reflecting complex and dynamic interactions between patterns of community awareness, service capacity, help seeking, and sociodemographic factors (3–7). In absolute terms, ASD affects 28.3 million people worldwide, and it is three to four times more prevalent in men than women (8). In Spain, rough estimates point to the existence of approximately half a million people of all ages with ASD (9, 10), but there is no official statistical data available.

Currently, available and official population databases in Spain consider only broad health categories such as developmental (11, 12) or mental disorders (13, 14), thereby providing highly inaccurate or outdated information related to autism. Other sources, like the last national health survey, reported an estimated prevalence of autism or ASD (0.6%) for the first time, though considering only children aged 3–14 and no further health data (15).

Cognitive and behavioral symptoms of ASD have a severe and life-long impact on the quality of life (QoL) and personal outcomes of people living with this condition (16); World Health Organization (2). Moreover, compared to the general population, premature mortality is at increased risk among autistic people due to their health comorbidities and other accidental factors (17–20). In this regard, more than 70% of people on the autism spectrum have some kind of neurological, gastrointestinal or immune co-occurring disorder, among others (21–23), and they are at significant risk of stroke, seizure and chronic diseases like obesity, diabetes, dyslipidemia, hypertension, coronary heart disease, and cancer (24, 25).

People with ASD are also more prone to mental health issues than neurotypicals (24, 26). A cohort-based study found that 70% of children and adolescents on the autism spectrum had one or more co-occurring mental health conditions, and 41% of them presented two or more (27). According to a recent meta-analysis, the most frequent ASD-associated mental health disorders in all ages are: Attention-deficit hyperactivity disorder (ADHD), anxiety disorder, sleep-wake disturbances, disruptive behaviors, impulse-control, and conduct disorder, depressive disorder, obsessive-compulsive disorder (OCD), bipolar disorder and those within the schizophrenia spectrum (26).

Several barriers and disadvantaging factors may compound the aforementioned ASD-related health disparities and limit people’s access to healthcare services (28, 29). Specifically, some studies suggest that the existence of concomitant ID and being a women predispose to a worse health profile (24, 26, 30–34). In addition, people on the autism spectrum may experience accelerated aging and age-related diseases at younger ages compared to the general population (32, 35).

Despite this evidence, most health problems beyond ASD-related symptoms have been overlooked for decades. Only a few studies have systematically addressed them in the last years (22, 26, 36), and fewer still have included adult individuals (24, 30, 33, 37). Thus, the lack of research on health outcomes is a significant barrier to promoting the QoL of people on the autism spectrum. It is also an obstacle to improving health care systems and developing evidence-based policies.

Here, we present the first Spanish epidemiologic study describing the health status of a large cohort of children, adolescents, and adults with ASD, including physical and mental health co-occurring conditions. We aim to analyse how their health is influenced by sex, age, and concurrent ID.

## 2. Materials and methods

### 2.1. Design and participants

We conducted an observational retrospective analytic study with a cohort of autistic individuals in Spain based on demographic and health data from a national registry collected by Autism Spain (38). Autism Spain is the leading charity related to ASD in Spain. It brings together 151 non-profitable organizations from all over the country that support people on the autism spectrum and their families to achieve equal opportunities and guarantee their QoL.

Autism Spain’s ASD registry contains information about people with a confirmed clinical diagnosis of ASD of any age. Most of them are users or members of autism supportive associations linked to Autism Spain and receive support from them (psychosocial, educational, occupational, juridical, and administrative, among others). The data collection period fell between the date of the first and last entries into the registry (November 2017 and May 2020).

**FIGURE 1:**
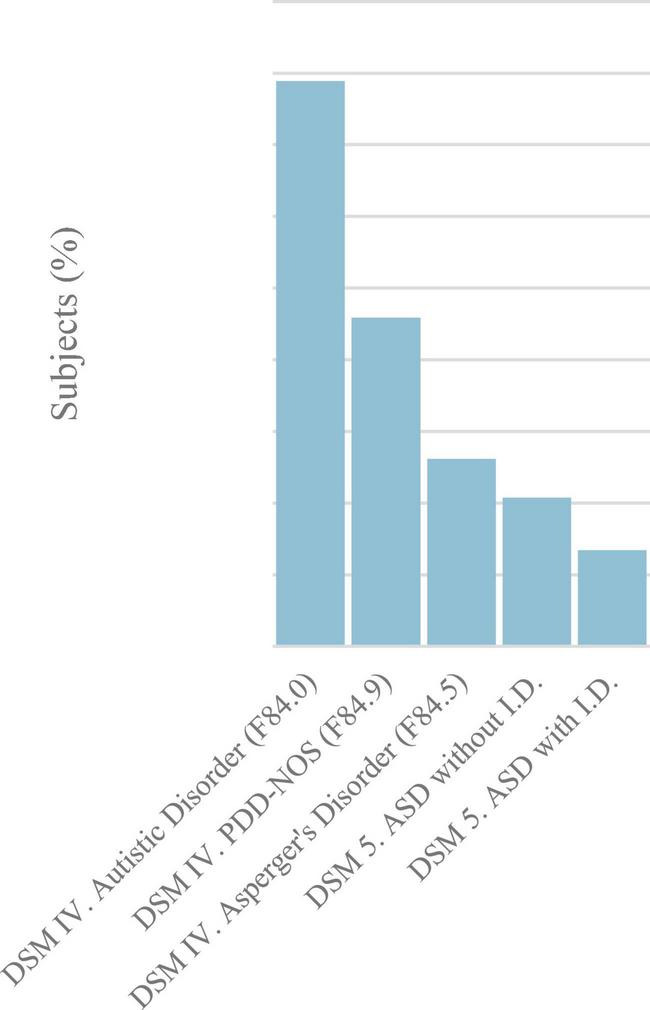
Distribution of study participants (*N* = 2,563) diagnosed according to DSM-IV or ICD-10 criteria for pervasive developmental disorder (PDD) and according to DSM 5 criteria for ASD. ASD, autism spectrum disorder; DSM-IV, American Psychiatric Association’s Diagnostic and Statistical Manual of Mental Disorders, Fourth Edition; DSM-5, American Psychiatric Association’s Diagnostic and Statistical Manual of Mental Disorders, Fifth Edition; ICD-10, International Classification of Diseases 10th Revision.

Forty-one ASD associations participated in this study and provided data related to 2,623 autistic people. Six participants contributed independently to the registry. Inclusion criteria were: (a) having a confirmed clinical diagnosis of any pervasive developmental disorder (PDD) (according to the fourth edition of the Diagnostic and Statistical Manual of Mental Disorders (DSM-IV), the International Classification of Diseases 10th Revision (ICD-10 or earlier diagnostic criteria) or ASD (according to the recent DSM fifth edition) (2, 16, 39), ICD-10 codes: F84.0, F84.2, F84.3, F84.5, F84.9 (Figure 1) and (b) providing informed consent to participate. Diagnoses from health care centers or authorized non-medical centers with ASD diagnostic services were included in the analysis. All registered individuals fulfilled these criteria and therefore provided data that were subsequently analysed. No drop-out events or experimental deaths were reported.

This study was performed in line with the principles of the Declaration of Helsinki. Approval was granted by the Ethics Committee of the Autonomous University of Madrid (CEI-105-2039). All individuals provided written informed consent to participate and allowed the publication of the results. Personal data were handled according to the current General Data Protection Regulation 2016/679 of the European Parliament (EU-GPDR) and the Council of 27 April 2016 and with the Spanish Organic Law 3/2018 of 5 December 2018 on Data Protection and Guarantee of Digital Rights. Due to the retrospective nature of this study, there was no influence on any medical decision, including treatment prescription.

### 2.2. Description of the variables and outcome measures

Data from the registries was analysed according to the following sociodemographic and health variables:

Sociodemographics

– Sex and age. Boys and girls and adolescents (age 18 or younger) and adults (older than 18).
– Health data. Health information is based on the clinical data included in health records and reports provided by the participants. Only those health conditions formally diagnosed by a specialized health practitioner (both in the Spanish public Health Care System or in private authorized health care services) according to international classification systems [DSM-IV (16; or ICD-10 (40)] were included in the registry.
– Diagnosis of any disorder within the autism spectrum (hereinafter “ASD”): according to DSM-IV (39), DSM-5 (16), or ICD-10 (2).
– Co*-*occurring conditions: divided into mental health disorders and other general medical conditions with a clinical confirmed diagnosis.
Those conditions with a stronger pathophysiological link to ASD, such as genetic (21) and nervous system disorders, (ICD-10 codes: G40.90, Q05, G80, P04.3), especially epilepsy (41–43), were grouped apart.
– Intellectual Disability*:* corresponding to an intelligence quotient (IQ) score <76 estimated with any standardized intelligence test (Weschler Adult Intelligence Scale IV, Wechsler Intelligence Scale for Children Revised and IV, Wechsler Preschool and Primary Scale of Intelligence III and IV, Wechsler Non-verbal Scale of Ability, Kaufman Brief Intelligence Test, Kaufman Assessment Battery for Children). ID level was then classified as profound (IQ < 20), severe (*IQ* = 20–40), moderate (*IQ* = 41–55), mild (*IQ* = 56–75), border (*IQ* = 76–85), or absent (IQ > 85), according to ICD-10 subcategories (2).
– Psychopharmacological treatment: only currently prescribed psychiatric drugs were considered.
– Functional disability: defined by a degree of general disability of ≥33% according to the official certificate issued by the Institute of Social Services and the Elderly (IMSERSO) of the Spanish Ministry of Social Rights and 2030 Agenda. This is a summary score of the individual’s functional limitations due to physical, mental, intellectual, or sensorial impairments. When reaching a percentage of ≥25%, social factors that limit equal, effective, and full participation in society, are added to the calculations. Those provided with a certificate equal or upper to a 33% percentage are eligible to apply for a government subsidy (44). This study rated functional disability in daily living skills according to the following percentage ranges: ≥75, 65–74, 33–64, and <33%.

Participants’ information was collected through a data entry questionnaire linked to the *Autism Spain’s* registry (available as Supplementary material). Affiliated members of *Autism Spain* had access only to their own registers. in each autism-support association the questionnaire was filled in by a designated professional that the Confederation had previously trained to contrast the information in the medical and social reports. The Confederation did not fix or save any copies of these documents.

Outcome measures in this study were the frequency of sociodemographic variables, clinical diagnosis, mental and physical health comorbidities, and psychiatric psychopharmacological prescription in the study population. Also, the distribution of health-related variables was analysed: neurological, chronic health diseases, and mental health disorders, as well as psychopharmacological treatment according to sex, age, and intellectual disability; diagnosis of ASD according to sex; ID level according to sex and age; and degree of functional disability according to sex and ID.

### 2.3. Data analysis

Participant data were analysed by descriptive statistics. Absolute and relative frequencies were used to describe categorical variables. The continuous ones were expressed by the mean and standard deviation (SD). Inferential tests were conducted to compare variables according to sex, age, and ID level: Student’s *t*-test (continuous variables) and Chi-square test (categorical variables). Statistical significance was set at *P* < 0.05. When possible, point-estimates of effect-size (odds ratio for variables with two categories and Cramér’s V for variables with more than two categories) with 95% confidence intervals (CI) for each inferential test were also conducted. Data analysis was performed by IBM’s Statistical Package for the Social Sciences (SPSS^®^) for Windows version 24 (IBM Corp. Released 2016. IBM SPSS Statistics for Windows, Version 24.0. Armonk, NY: IBM Corp.).

## 3. Results

### 3.1. Sociodemographics of the study sample

*Autism Spain’s* registry contained information about 2,629 individuals with a confirmed diagnosis of ASD. Mean age was significantly higher for women than for men (18.4; SD = 12.1 vs. 15.8; SD = 10.3, respectively, *P* < 0.0001). Almost one third of the study population were adults (*N* = 798; 30.4%), and approximately half of them (*N* = 424; 16.2%) were older than 25 and up to 60 years old (see Table 1).

**TABLE 1:** Sociodemographic characteristics of autism spectrum disorder (ASD).

	Total subjects
Sex (male), *n* (%)	2,158 (82.1)
**Age, years^1^**	
Mean (SD)	16.2 (10.7)
**Age range, *n* (%)^1^**	
≤18 years (non-adults)	1,825 (69.6)
>18 years (adults)	798 (30.4)
0–5 years	312 (11.9)
6–11 years	779 (29.7)
12–17 years	734 (28.0)
18–25 years	374 (14.3)
>25 years	424 (16.2)

Unless otherwise indicated, percentages refer to the total study population (*N* = 2,629).

^1^*N* = 2,623; *n* missing = 6.

### 3.2. Autism spectrum disorder diagnosis

**FIGURE 2:**
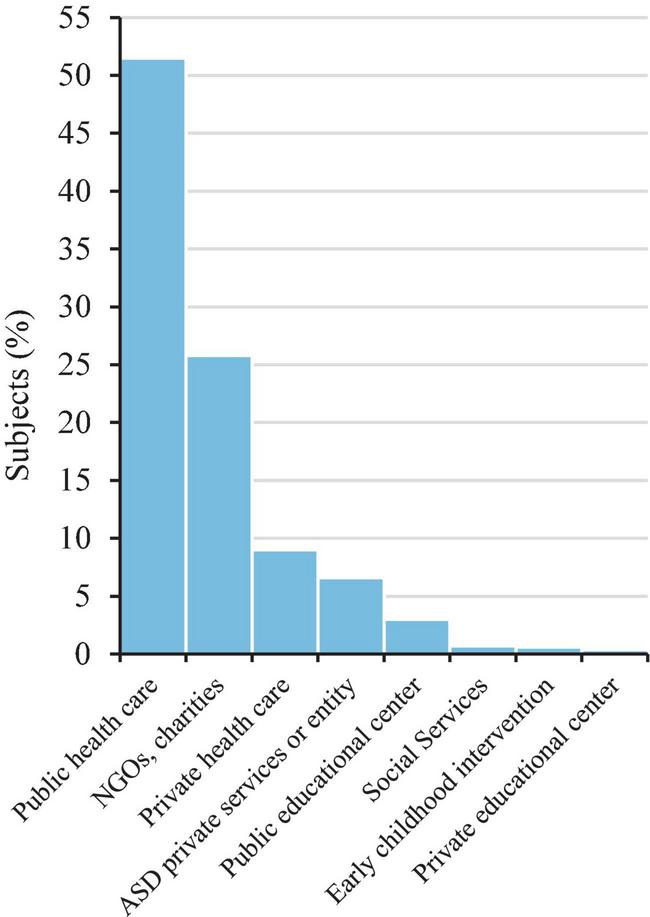
Location of diagnosis of the study participants (*N* = 2,563). NGO, non-governmental organization.

**FIGURE 3:**
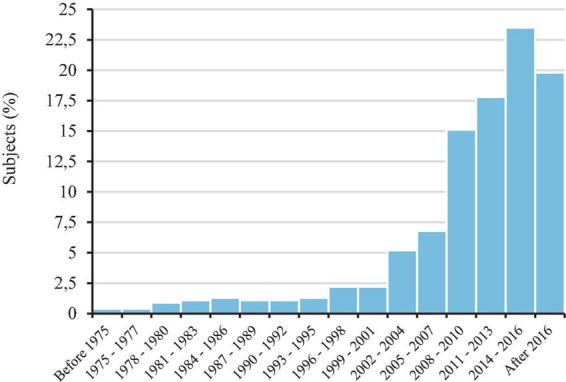
Percentages of autism spectrum disorder (ASD) diagnoses between before 1975 and after 2016.

We found that mean age at diagnosis time was 7.5 years (SD = 6.5), and almost all participants (*N* = 2,344; 95.8%) had received it before age 21. Public health care (*N* = 1,320; 51.5%) and diagnostic services at non-governmental organizations related to ASD (*N* = 662; 25.8%) were the most common diagnostic sites (Figure 2). The number of diagnoses showed an increasing trend during the last two decades and especially between the 2005–2007 (*N* = 167; 6.8%) and the 2008–2010 (*N* = 370; 15.1%) periods (see Figure 3).

### 3.3. Occurrence of intellectual disability and recognition of supporting needs

**FIGURE 4:**
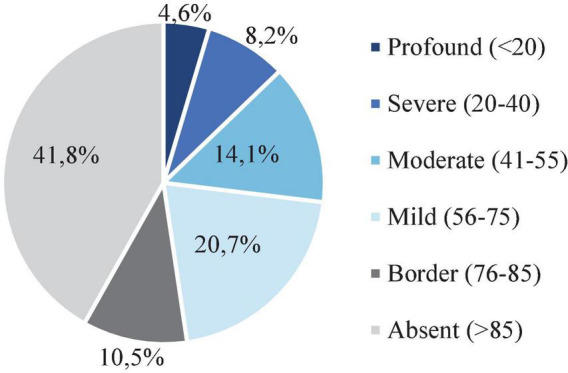
Distribution of intellectual disability severity degree of the study participants. The level of intellectual disability (ID) according to the intelligence quotient (IQ) test score. ID corresponds to an IQ test score <76.

Around a third of the study participants (*N* = 885; 33.7%) had been tested with an IQ formal test. Mean IQ score was 76.1 (SD = 30.9) for the whole study population, and it was higher in men than in woman (78.2; SD = 30.3 vs. 66.8; SD = 32.0, respectively, *P* < 0.0001). Approximately half of the individuals had no ID accompanying the ASD diagnosis (ICD-10 Codes: F70, F71. F72, F73, F78, and F79) (*N* = 463; 52.3%), while the other half (*N* = 422; 47.7%) presented this concurrent condition in different severity levels (Figure 4).

Adaptative skills had been officially assessed for most of the study population (*N* = 2,219; 84.4%). In most cases some degree of functional impairment that provided access to public support was identified (*N* = 2,181; 98.3%).

### 3.4. Genetic and nervous system disorders, other co-occurring physical and mental conditions, and psychopharmacological treatment

Among those with any confirmed genetic disorders (*N* = 134; 5.1%), Fragile X Syndrome (ICD-10 code: Q99.2) (*N* = 9; 0.3%) and Down Syndrome (ICD-10 codes: Q90.X) (*N* = 7; 0.3%) were the most frequent ones (Table 2). Other identified genetic disorders (*N* = 118; 4.5%) corresponded to a wide range of distinct chromosomic anomalies. A total of 338 (12.9%) individuals had a nervous system disorder. Epilepsy and recurrent epileptic seizures (ICD-10 codes G40.X) (*N* = 288; 11.0%) were the most frequently reported in this category (see Table 2).

**TABLE 2:** Genetic, nervous system, and other comorbid disorders.

	Total subjects (*N* = 2,629)
**Genetic disorders, *n* (%)**	
Fragile X syndrome (Q99.2)	9 (0.3)
Don syndrome (Q90.X)	7 (0.3)
**Nervous system disorders, *n* (%)**	
Epilepsy and recurrent epileptic seizures (G40.X)	288 (11.0)
Spina bifida (Q05.X) and other congenital anomalies of the nervous system (Q00-07)	25 (1.0)
Cerebral palsy (G 80.X)	11 (0.4)
Fetal alcohol syndrome (Q86.0)	5 (0.2)
**Mental health disorders, *n* (%)**	
Impulse-control and disruptive behavior disorder (F63)	304 (11.6)
Anxiety disorder (F41)	179 (6.8)
Attention deficit and hyperactivity disorder (F90.X)	151 (5.8)
Obsessive compulsive disorder (F42.X)	117 (4.5)
Eating disorders (F50.X)	109 (4.1)
Depressive disorders (F32.X)	75 (2.9)
Personality disorders (F60.X)	22 (0.8)
Phonological disorder (R47.X)	12 (0.5)
**General medical conditions, *n* (%)**	
Sleep-wake disturbances (F51.3)	175 (6.7)
Dermatitis and eczema (L30.9)	123 (4.7)
Overweight and obesity (E66.X)	111 (4.2)
Intestinal disorders (K55-K63)	107 (4.1)
Dentofacial anomalies and other mandibular disorders (M26-M27)	43 (1.6)
Thyroid gland disorders (E03.X, E05.X)	36 (1.4)
Gastric ulcer (K25.X), gastritis (K29.0-7) or esophagitis (K20.X)	20 (0.8)
Visual problems (H53-H54)	21 (0.8)
Other metabolic disorders (E70-E90)	19 (0.7)
Vasomotor (J30.0) and allergic rhinitis (J30.9)	17 (0.6)
Hearing loss (H90-H91)	14 (0.5)
Kidney disease (N18.X) or other affected urinary tract system organs (N30-N39)	14 (0.5)
Diabetes (E08-E013)	12 (0.5)
Urticaria (L50.X)	8 (0.3)

International Classification of Diseases 10th Revision (ICD-10) codes between brackets.

**FIGURE 5:**
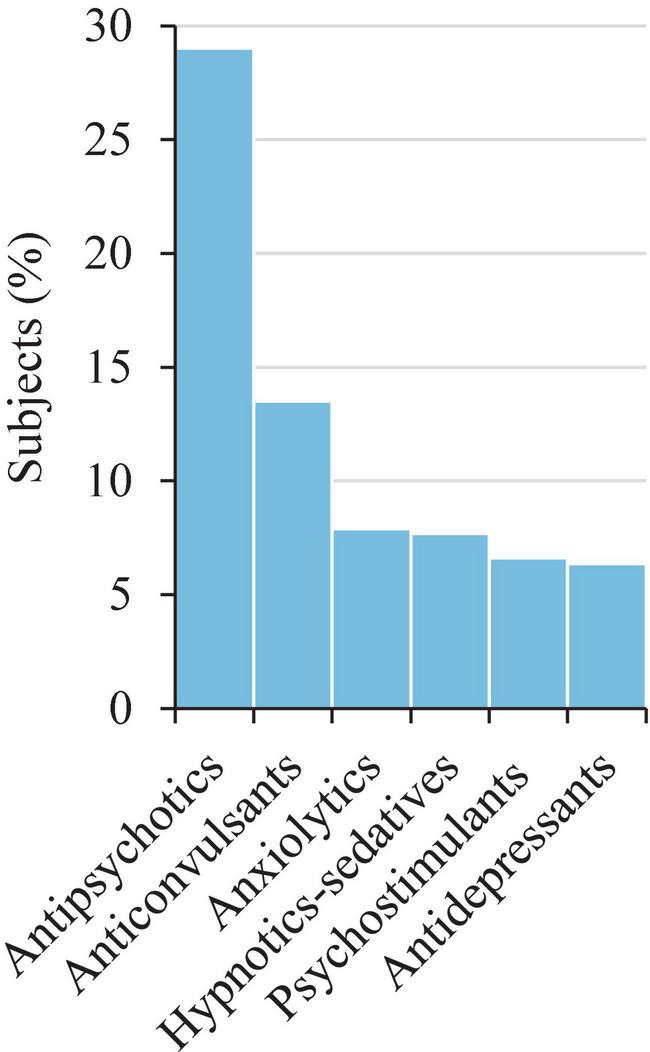
Current psychopharmacological treatment: therapeutic groups. Only those with ≥ 5% of users are shown (*N* = 2,629). Antipsychotics (risperidone, aripiprazole), anticonvulsants (valproates, sulfamates, carboxamides), anxiolytics (benzodiazepines), hypnotic-sedatives (melatonin), psychostimulants (methylphenidate), and antidepressants (SSRIs). Only those with ≥ 1% of users are shown (*N* = 2,629). SSRIs, selective serotonin reuptake inhibitors.

Around one fifth (*N* = 467; 17.8%) of the studied sample had one or more mental health diagnoses apart from ASD with or without ID, namely: impulse-control and disruptive behavior disorder (ICD-10 code: F63) (*N* = 304; 11.6%), anxiety disorder (ICD-10 code: F41) (*N* = 179; 6.8%), attention deficit and hyperactivity disorder (ICD-10 code: F90.X) (*N* = 151; 5.8%), obsessive compulsive disorder (ICD-10 codes: F42.X) (*N* = 117; 4.5%), and eating disorders (ICD-10 codes: F50.X) (*N* = 109; 4.1%), among others (see Table 2). Almost half (*N* = 1,187; 45.2%) of the participants were currently taking psychopharmacological drugs, mainly antipsychotics (*N* = 763; 29.0%) and anticonvulsants (*N* = 355; 13.5%) (Figure 5).

Other general medical conditions were found in 668 (25.4%) people. In some cases, two (*N* = 138; 5.2%), three (*N* = 53; 2.0%), or more than three (*N* = 33; 1.3%) comorbidities were reported for the same individual. The main ones were sleep-wake disturbances (ICD-10 code: F51.3) (*N* = 175; 6.7%), dermatitis and eczema (ICD-10 code: L30.9) (*N* = 123; 4.7%), overweight and obesity (ICD-10 code: E66.X) (*N* = 111; 4.2%), and intestinal disorders (ICD-10 codes: K55-K63) (*N* = 107; 4.1%) (see Table 2).

### 3.5. Health disparities according to sex, age, and cognitive functioning

Women presented more severe forms of ID (moderate-to-severe levels) than the male subgroup (*P* = 0.0002; Table 3). The degree of functional impairment in daily living skills was also differently distributed according to sex (*P* < 0.0001). The percentage of women with this type of difficulties almost doubled that of men (see Table 3).

**TABLE 3:** Intellectual and functional disability, nervous system, and mental health disorders, general medical conditions and psychopharmacological treatment according to sex.

	Men	Women	Size effects: Cramer’s V or OR (IC 95%)	*P*-value
Level of ID according to the IQ test score, *n* (%)	724 (100.0)	161 (100.0)	0.158	0.0002
Profound (<20)	28 (3.9)	13 (8.1)	–	–
Severe (20–40)	56 (7.7)	17 (10.6)	–	–
Moderate (41–55)	89 (12.3)	36 (22.4)	–	–
Mild (56–75)	152 (21.0)	31 (19.3)	–	–
Border (76–85)	79 (10.9)	14 (8.7)	–	–
Absent (>85)	320 (44.2)	50 (31.1)	–	–
Degree of functional disability, *n* (%)	1,820 (100.0)	399 (100.0)	0.147	<0.0001
<33%	32 (1.8)	6 (1.5)	–	–
33–64%	1,059 (58.2)	174 (43.6)	–	–
65–74%	297 (16.3)	57 (14.3)	–	–
≥75%	432 (23.7)	162 (40.6)	–	–
Nervous system disorders, *n* (%)	2,158 (100.0)	471 (100.0)	–	–
Individuals with ≥1 nervous system disorder	242 (11.2)	96 (20.4)	0.49 (0.38–0.64)	<0.0001
Epilepsy and recurrent epileptic seizures (G40.X)	205 (9.5)	83 (17.6)	0.49 (0.37–0.65)	<0.0001
Spina bifida (Q05.X) and other congenital anomalies of the nervous system (Q00-07)	16 (0.7)	9 (1.9)	0.38 (0.17–0.87)	0.0178
Individuals who do not declare nervous system disorders	1,916 (88.8)	375 (79.6)	–	<0.0001
Mental health disorders, *n* (%)	2,158 (100.0)	471 (100.0)	–	–
Individuals with ≥1 mental health disorder	532 (24.7)	136 (28.9)	0.81 (0.65–1.01)	0.0565
Number	2,158 (100.0)	471 (100.0)	0.06	0.048
0	1,626 (75.3)	335 (71.1)	–	–
1	362 (16.8)	85 (18.0)	–	–
2	106 (4.9)	27 (5.7)	–	–
3	35 (1.6)	17 (3.6)	–	–
>3	29 (1.3)	7 (1.5)	–	–
Type	–	–	–	–
Impulse-control and disruptive behaviour disorders (F63)	232 (10.8)	72 (15.3)	0.67 (0.50–0.89)	0.0053
Anxiety disorders (F41)	131 (6.1)	48 (10.2)	0.57 (0.40–0.81)	0.0013
Obsessive compulsive disorder (F42.X)	93 (4.3)	24 (5.1)	0.84 (0.53–1.33)	0.4536
Eating disorders (F50.X)	87 (4.0)	22 (4.7)	0.86 (0.53–1.38)	0.5283
Depressive disorders (F32.X)	52 (2.4)	23 (4.9)	0.48 (0.29–0.79)	0.0035
Others	210 (9.7)	32 (6.8)	1.48 (1.01–2.18)	0.0458
Attention deficit and hyperactivity disorder (F90.X)	136 (64.8)	15 (46.9)	2.04 (1.19–3.52)	–
Personality disorders (F60.X)	19 (9.0)	3 (9.4)	1.39 (0.41–4.70)	–
Phonological disorder (R47.X)	11 (5.2)	1 (3.1)	2.41 (0.31–18.7)	–
General medical conditions, *n* (%)	2,158 (100.0)	471 (100.0)	–	–
Individuals with ≥1 comorbid condition	465 (21.5)	155 (32.9)	0.56 (0.45–0.70)	<0.0001
Sleep-wake disturbances (F51.3)	126 (5.8)	49 (10.4)	0.53 (0.38–0.75)	0.0003
Overweight and obesity (E66.X)	79 (3.7)	32 (6.8)	0.52 (0.34–0.80)	0.0022
Intestinal disorders (K55-K63)	78 (3.6)	29 (6.2)	0.57 (0.37–0.89)	0.0114
Dentofacial anomalies and other mandibular disorders (M26-M27)	28 (1.3)	15 (3.2)	0.40 (0.21–0.75)	0.0034
Individuals who do not declare general medical conditions	1693 (78.4)	316 (67.1)	–	0.0001
Psychopharmacological treatment, *n* (%)	2,158 (100.0)	471 (100.0)	–	–
Individuals currently prescribed with ≥1 psychiatric drug	950 (44.0)	237 (50.3)	0.78 (0.64–0.95)	0.0129
Antipsychotics	627 (29.1)	136 (28.9)	1.01 (0.81–1.26)	0.9379
Anticonvulsants	262 (12.1)	93 (19.7)	0.56 (0.43–0.73)	<0.0001
Anxiolytics	161 (7.5)	47 (10.0)	0.73 (0.52–1.02)	0.0666
Melatonin	149 (6.9)	42 (8.9)	0.76 (0.53–1.08)	0.1273
Antidepressants	133 (6.2)	34 (7.2)	0.84 (0.57–1.25)	0.3948
Methylphenidate	121 (5.6)	16 (3.4)	1.69 (0.99–2.87)	0.0506

Condition of disability is defined by a degree of functional disability of ≥33%. Condition of ID corresponds to an IQ test score <76. OR only shown for dichotomous variables. International Classification of Diseases 10th Revision (ICD-10) codes between brackets.

Proportionally, more women than men endured one or more nervous system disorders (OR = 0.49; 95% CI = 0.38–0.64; *P* < 0.0001) and other medical conditions (OR = 0.56; 95% CI = 0.45-0.70; *P* < 0.0001). Mental health co-occurring disorders showed the same trend, although not reaching statistical significance (OR = 0.81; 95% CI = 0.65–1.01; *P* = 0.0565), and they also received more psychopharmacological treatments than men (OR = 0.78; 95% CI = 0.64-0.95; *P* = 0.0129) (see Table 3).

The occurrence of multiple nervous system (*P* < 0.0001), mental health (*P* < 0.0001), and other health comorbid disorders (*P* < 0.0001) as well as the number of individuals with psychopharmacological prescriptions (*P* < 0.0001) increased with age. However, the prescription of melatonin (*P* < 0.0001) and methylphenidate (*P* < 0.0001) showed the reverse tendency, reaching its highest in infancy and childhood, and childhood and adolescence, respectively, (see Table 4).

**TABLE 4:** Intellectual disability, nervous system, and mental health disorders, general medical conditions and psychopharmacological treatment according to age groups.

	0–5 years	6–11 years	12–17 years	18–25 years	>25 years	*P*-value
Level of ID according to the IQ test score, *n* (%)	54 (100.0)	249 (100.0)	290 (100.0)	137 (100.0)	154 (100.0)	<0.0001
Profound (<20)	2 (3.7)	4 (1.6)	5 (1.7)	7 (5.1)	23 (14.9)	–
Severe (20–40)	–	7 (2.8)	11 (3.8)	11 (8.0)	44 (28.6)	–
Moderate (41–55)	13 (24.1)	26 (10.4)	36 (12.4)	24 (17.5)	26 (16.9)	–
Mild (56–75)	17 (31.5)	49 (19.7)	73 (25.2)	29 (21.2)	15 (9.7)	–
Border (76–85)	7 (13.0)	32 (12.9)	35 (12.1)	10 (7.3)	9 (5.8)	–
Absent (>85)	15 (27.8)	131 (52.6)	130 (44.8)	56 (40.9)	37 (24.0)	–
Nervous system disorders, *n* (%)	312 (100.0)	779 (100.0)	734 (100.0)	374 (100.0)	424 (100.0)	–
Individuals with ≥1 nervous system disorder	9 (2.9)	54 (6.9)	71 (9.7)	48 (12.8)	156 (36.8)	<0.0001
Epilepsy and recurrent epileptic seizures (G40.X)	6 (1.9)	41 (5.3)	56 (7.6)	43 (11.5)	142 (33.5)	<0.0001
Individuals who do not declare nervous system disorders	303 (97.1)	725 (93.06)	663 (90.3)	326 (87.2)	268 (63.2)	<0.0001
Mental health disorders, *n* (%)	312 (100.0)	779 (100.0)	734 (100.0)	374 (100.0)	424 (100.0)	–
Individuals with ≥1 mental health disorder	27 (8.7)	147 (18.9)	186 (25.3)	106 (28.3)	200 (47.2)	<0.0001
Number	312 (100.0)	779 (100.0)	734 (100.0)	374 (100.0)	424 (100.0)	<0.0001
0	285 (91.3)	632 (81.1)	548 (74.7)	268 (71.7)	224 (52.8)	
1	19 (6.1)	120 (15.4)	135 (18.4)	69 (18.4)	103 (24.3)	
2	5 (1.6)	17 (2.2)	34 (4.6)	20 (5.3)	56 (13.2)	
3	3 (1.0)	7 (0.9)	9 (1.2)	8 (2.1)	25 (5.9)	
>3	–	3 (0.4)	8 (1.1)	9 (2.4)	16 (3.8)	
Type	–	–	–	–	–	–
Impulse-control and disruptive behaviour disorders (F63)	10 (3.2)	56 (7.2)	65 (8.9)	45 (12.0)	127 (30.0)	<0.0001
Anxiety disorders (F41)	3 (1.0)	25 (3.2)	39 (5.3)	38 (10.2)	73 (17.2)	<0.0001
Obsessive compulsive disorder (F42.X)	6 (1.9)	13 (1.7)	24 (3.3)	22 (5.9)	52 (12.3)	<0.0001
Depressive disorders (F32.X)	–	3 (0.4)	13 (1.8)	15 (4.0)	43 (10.1)	<0.0001
Others	6 (100.0)	62 (100.0)	99 (100.0)	34 (100.0)	41 (100.0)	<0.0001
Phonological disorder (R47.X)	2 (33.3)	5 (8.1)	4 (4.0)	1 (2.9)	–	
Attention deficit and hyperactivity disorder (F90.X)	1 (16.7)	43 (69.4)	73 (73.7)	23 (67.6)	11 (26.8)	
Personality disorders (F60.X)	–	1 (1.6)	4 (4.0)	4 (11.8)	13 (31.7)	
General medical conditions, *n* (%)	312 (100.0)	779 (100.0)	734 (100.0)	374 (100.0)	424 (100.0)	–
Individuals with ≥ 1 comorbid condition	44 (14.1)	161 (20.7)	130 (17.7)	74 (19.8)	211 (49.8)	<0.0001
Sleep-wake disturbances (F51.3)	14 (4.5)	38 (4.9)	31 (4.2)	18 (4.8)	74 (17.5)	<0.0001
Overweight and obesity (E66.X)	–	12 (1.5)	18 (2.5)	21 (5.6)	60 (14.2)	<0.0001
Intestinal disorders (K55-K63)	12 (3.8)	28 (3.6)	29 (4.0)	9 (2.4)	29 (6.8)	0.0217
Dentofacial anomalies and other mandibular disorders (M26-M27)	3 (1.0)	13 (1.7)	8 (1.1)	2 (0.5)	17 (4.0)	0.0005
Thyroid gland disorders (E03.X, E05.X)	2 (0.6)	10 (1.3)	6 (0.8)	4 (1.1)	14 (3.3)	0.005
Gastric ulcer (K25.X), gastritis (K29.0-7) or esophagitis (K20.X)	1 (0.3)	4 (0.5)	3 (0.4)	2 (0.5)	10 (2.4)	0.0018
Metabolic disorders (E70-E90)	1 (0.3)	7 (0.9)	2 (0.3)	1 (0.3)	8 (1.9)	0.0161
Individuals who do not declare general medical conditions	–	–	–	–	–	–
Psychopharmacological treatment, *n* (%)	312 (100.0)	779 (100.0)	734 (100.0)	374 (100.0)	424 (100.0)	–
Individuals currently prescribed with ≥1 psychiatric drug	64 (20.5)	300 (38.5)	333 (45.4)	180 (48.1)	306 (72.2)	<0.0001
Antipsychotics	20 (6.4)	174 (22.3)	207 (28.2)	127 (34.0)	233 (55.0)	<0.0001
Anticonvulsants	7 (2.2)	34 (4.4)	74 (10.1)	76 (20.3)	163 (38.4)	<0.0001
Anxiolytics	1 (0.3)	6 (0.8)	22 (3.0)	36 (9.6)	142 (33.5)	<0.0001
Melatonin	37 (11.9)	79 (10.1)	44 (6.0)	18 (4.8)	13 (3.1)	<0.0001
Antidepressants	–	12 (1.5)	41 (5.6)	43 (11.5)	70 (16.5)	<0.0001
Methylphenidate	3 (1.0)	51 (6.5)	69 (9.4)	8 (2.1)	6 (1.4)	<0.0001
Individuals who do not declare psychopharmacological treatment	289 (92.6)	554 (71.1)	451 (1.4)	225 (60.1)	146 (34.4)	<0.0001

Condition of ID corresponds to an IQ test score <76. ID, intellectual disability; IQ, intelligence quotient; OCD, obsessive-compulsive disorder. International Classification of Diseases 10th Revision (ICD-10) codes between brackets.

Intellectual disability was associated with a greater risk of neurological disorders (OR = 0.16; 95% CI = 0.10–0.26; *P* < 0.0001) and other medical co-occurring conditions (OR = 0.35; 95% CI = 0.26–0.48; *P* < 0.0001) (see Table 5). Mental health comorbidities impacted differently depending on the ID level: impulse-control and disruptive behavior (OR = 0.30; 95% CI = 0.19–0.47; *P* < 0.0001) and OCD (OR = 0.39; 95% CI = 0.19–0.80; *P* = 0.0076) were more common in the group with ID, whereas ADHD and personality disorders had been diagnosed to a greater extent among those without ID (OR = 2.14; 95% CI = 1.46–3.13; *P* < 0.0001) (Table 5). Psychopharmacological drug prescription was generally higher in the ID group (OR = 0.34; 95% CI = 0.26–0.45; *P* < 0.0001) except for methylphenidate, which showed the reverse tendency (OR = 3.51; 95% CI = 1.95–6.32) (Table 5).

**TABLE 5:** Functional disability, nervous system, and mental health disorders, general medical conditions and psychopharmacological treatment according to intellectual quotient groups.

	IQ ≥ 76	IQ < 76	Size effects: Cramer’s V or OR (IC 95%)	*P*-value
Degree of functional disability, *n* (%)	352 (100.0)	399 (100.0)	0.117	<0.0001
<33%	22 (6.3)	1 (0.3)	–	–
33–64%	280 (79.5)	162 (40.6)	–	–
65–74%	43 (12.2)	67 (16.8)	–	–
≥75%	7 (2.0)	169 (42.4)	–	–
Nervous system disorders, *n* (%)	463 (100.0)	422 (100.0)	–	–
Individuals with ≥1 nervous system disorder	20 (4.3)	94 (22.3)	0.16 (0.10–0.26)	<0.0001
Epilepsy and recurrent epileptic seizures G40.X)	11 (2.4)	85 (20.1)	0.10 (0.05–0.18)	<0.0001
Mental health disorders, *n* (%)	463 (100.0)	422 (100.0)	–	–
Individuals with ≥1 mental health disorder	141 (30.5)	134 (31.8)	0.94 (0.71–1.25)	0.6764
Type	–	–	–	–
Impulse-control and disruptive behavior disorders (F63)	27 (5.8)	73 (17.3)	0.30 (0.19–0.47)	<0.0001
Obsessive compulsive disorder (F42.X)	11 (2.4)	25 (5.9)	0.39 (0.19–0.80)	0.0076
Others	96 (100.0)	46 (100.0)	2.14 (1.46–3.13)	<0.0001
Attention deficit and hyperactivity disorder (F90.X)	73 (76.0)	25 (54.3)	2.97 (1.85–4.78)	–
Personality disorders (F60.X)	10 (10.4)	4 (8.7)	2.31 (0.72–7.41)	–
General medical conditions, *n* (%)	463 (100.0)	422 (100.0)	–	–
Individuals with ≥1 comorbid condition	85 (18.4)	165 (39.1)	0.35 (0.26–0.48)	<0.0001
Overweight and obesity (E66.X)	17 (3.7)	31 (7.3)	0.48 (0.26–0.88)	0.0159
Sleep-wake disturbances (F51.3)	12 (2.6)	60 (14.2)	0.16 (0.09–0.30)	<0.0001
Intestinal disorders (K55-K63)	9 (1.9)	31 (7.3)	0.25 (0.12–0.53)	0.0001
Dentofacial anomalies and other mandibular disorders (M26-M27)	4 (0.9)	19 (4.5)	0.18 (0.06-0.55)	0.0007
Gastric ulcer (K25.X), gastritis (K29.0-7) or esophagitis (K20.X)	0 (0.0)	8 (1.9)	–	0.0029
Psychopharmacological treatment, *n* (%)	–	–	–	–
Individuals currently prescribed with ≥ 1 psychiatric drug	149 (32.2)	246 (58.3)	0.34 (0.26–0.45)	<0.0001
Antipsychotics	68 (14.7)	172 (40.8)	0.25 (0.18–0.35)	<0.0001
Methylphenidate	53 (11.4)	15 (3.6)	3.51 (1.95–6.32)	<0.0001
Anticonvulsants	16 (3.5)	96 (22.7)	0.12 (0.07–0.21)	<0.0001
Melatonin	13 (2.8)	33 (7.8)	0.34 (0.18–0.66)	0.0008
Anxiolytics	12 (2.6)	50 (11.8)	0.20 (0.10-0.38)	<0.0001

Condition of disability is defined by a degree of functional disability of ≥33%. Condition of intellectual disability (ID) corresponds to an IQ test score <76. OR only shown for dichotomous variables. CI, confidence interval; IQ, intelligence quotient; OCD, obsessive-compulsive disorder; OR, odds ratio. International Classification of Diseases 10th Revision (ICD-10) codes between brackets.

Finally, we found that the levels of intellectual and functional disabilities were highly correlated (*P* < 0.0001). More individuals with ID had been certified with the highest percentages of disability in proportion to those without ID (Table 5).

## 4. Discussion

For the first time in our country, we have characterized the health status of the largest autism dataset in Spain. We found that the overall health status of people with ASD in Spain is compromised by different factors. The mean age at ASD diagnosis was 7.5 years old and it was generally obtained through the public health system or non-governmental organizations specialized in autism care. Up to 25% of the sample presented nervous system disorders, mental health associated conditions and other comorbidities apart from ASD. Around 50% of them received psychopharmacological treatment. Being a woman, advanced age and the presence of ID were identified as potential exacerbating factors of health problems. Also, those with ID had poorer adaptive functioning, and women were more prone to severe intellectual and functional impairment.

### 4.1. Autism diagnosis

The average age at ASD diagnosis of the individuals in our sample corresponds to the estimated ranges in Europe between 1990 and 2012, which varied from 3 to 10 years of age. However, most up-to-date studies show that in recent years (2012–2019) it has decreased and oscillates rather between 3.5 and 5 years (45). Therefore, according to our data, it seems that in Spain age at diagnosis is still relatively late compared to the European standard.

A men-to-women ratio of 4:1 was observed in our sample, in line with traditional epidemiologic reports (46, 47). However, in the last decades, estimates have pointed to a reduced 3:1 ratio (8, 48, 49). Several authors have suggested the presence of a sex bias in ASD diagnosis, including more wrong, delayed or missed diagnoses in autistic women (50–52). This could be partially explained by a male-biased understanding of the condition, the existence of sex differences in clinical presentations and the lack of instruments and procedures to effectively recognize them (53, 54). Consequently, the prevalence of ASD in women may still be underestimated, also in the Spanish context.

### 4.2. Health comorbidities

Only a couple of genetic syndromes (Down and Fragile X) were identified, which explained less than 1% of the cases in our study. Recent evidence suggests that complex polygenic mechanisms and environmental factors contribute approximately the same to the etiology of autism (55, 56).

Regarding other pathologies accompanying ASD, we suspect that the prevalence of nervous system disorders, mental health diagnoses and other comorbidities in our sample may be underestimated since it falls far below that of other countries (around 70%) (21–23, 57). This may be due to considerable differences in health care systems or to the study design criteria (we only considered comorbid clinical diagnoses confirmed by a health care professional according to DSM or ICD international classification systems).

However, the categories found in greater representation in this study do correspond to those usually reported by other European studies on the health of people with autism (3, 58).

### 4.3. Pharmacological prescriptions

Regarding pharmacological treatments, we found that psychiatric polypharmacy was significantly higher in participants with ASD and ID, as well as in the women and elderly subgroups. In addition, the number of individuals with psychopharmacological prescriptions was higher than the total number of neurological and psychiatric confirmed diagnoses that require drug administration. Also, we noticed a sudden rise in the number of children (≥6 years old) under psychotropic treatment that was not mirrored by in the number of mental health co-occurring confirmed diagnoses. Likewise, the higher proportion of youngsters treated with methylphenidate than the actual number of ADHD cases in the sample is as well intriguing.

According to international guidelines and recommendations, antipsychotics, antidepressants, and anticonvulsants should not be prescribed to manage any core autism symptomatology (59). A case-by-case analysis should be performed to exclude or confirm any unnecessary or inappropriate interventions with those psychiatric medications. Instead, their use should be restricted to cases of concerning behaviors, i.e., when it jeopardizes QoL and/or safety (self or others’) (60, 61). Despite these recommendations, some authors claim that people with autism, ID or both, are currently overprescribed, especially with antipsychotics and antidepressants (62, 63), which may have deleterious effects on their physical and mental health throughout their lifespan (64). Our study results also point to this presumed overprescription of psychopharmacological treatments that increase with age and, possibly, in the absence of the indicated clinical conditions too. In this regard, rethinking psychiatric prescription protocols is imperative, perhaps by encouraging evidence-based psycho-educative practices, complementary or alternative to psychopharmacological prescriptions, and promoting good practices in their follow-up.

### 4.4. Poor health risk factors related to autism: Sex, age, intellectual disability

As in the present study, the ASD-associated medical conditions identified so far belong to a wide range of medical areas and are subject to both sex- and age-related disparities (24, 30, 33). Compared to the men ASD subpopulation, we observed that women were significantly more prone to neurological or other health disorders (epilepsy, spina bifida, sleep-wake disturbances, intestinal disorders, overweight, obesity, and dental abnormalities) and most likely to one or more mental health co-occurring conditions too (particularly, impulse control and conduct disorders, anxiety, and depression). Several reasons may explain these sex-based differences, such as the delays or errors in ASD diagnosis mentioned above and thus a lack of understanding and support for their needs (50–52). Other physiological factors such as hormonal imbalances, which may imply severe physical and mental complications for women, could also intervene (31, 34), but more research is needed to achieve solid conclusions. Finally, being a woman was also associated with higher ID levels and more complex support needs. Those women with milder supporting needs are less represented in our sample, as they are in similar studies (3, 65). Research on their reality and priorities should increase, in order to improve their QoL and the community response to their needs.

On the other hand, most of the analysed cooccurring conditions showed, as expected, an increasing trend with age. The percentage of individuals with more than one neurological disorder, mental health disorder or general condition already doubled or tripled in the >25 years-old group compared to younger ages. Considering that no adults in our study population were older than 60, our findings led us to suspect premature comorbidity onset in ASD compared to the general population. In connection to this result, emerging studies have suggested an association between ASD and accelerated aging after reporting the early onset of age-related disorders such as seizure, hypertension, hyperlipidemia, and chronic kidney disease, especially in the presence of ID. This health status decline is accompanied by progressive less autonomy, poorer adaptive skills and the use of polypharmacy (32, 35).

Mental health issues can, as well, profoundly affect QoL at younger ages. According to a recent European longitudinal study, depression and anxiety symptoms in children and adolescents with ASD significantly reduced their perception of wellbeing (66). The same age group is at an increased risk of an anxiety disorder (67, 68), which is even higher in the presence of ADHD (67). Reciprocally, the coexistence of ADHD and anxiety has been related to poorer adaptive skills in autistic children (67). In our results, we observed that school- and high school-stage ASD participants had already received a diagnosis of anxiety, although its prevalence increased at older ages together with the prescription of antidepressants and anxiolytics. On the contrary, ADHD was more frequently reported in children, which may be due to elder individuals’ under- or delayed diagnosis (69).

The risk for health-related issues also depends on the presence of ID (70, 71). Global estimates on the prevalence of cognitive impairment in ASD greatly vary across publications. The percentage found in our study (47.7% of tested individuals) falls between the estimated range of 40–61% (72), although newer evidence suggests that it can go down to 30% (31). Few studies have addressed the influence of ID on the health status of ASD individuals. In the meta-analysis performed by Lai and colleagues, heterogeneity in prevalence estimates of mental comorbidities in ASD was associated with intellectual functioning, besides other variables (26). Another publication of the same year found a high physical and mental comorbidity burden in adults with ASD and ID (mean age = 42.9) that was comparable to that of the general and older geriatric population (mean age = 79) from the same hospital (32). In our comparative analysis, an extensive range of physical and mental comorbidities were associated with the presence of ID, except for personality disorders and ADHD, possibly because they are challenging to diagnose in this context.

#### 4.4.1. Study limitations

Due to the retrospective nature of our research, there are some related limitations that must be taken into account when interpreting the results.

Although only clinically confirmed health issues according to international classifications had been included, changes in diagnosis criteria of those coexistent conditions over time and missing data could have biased our results.

Also, the sample representativeness is limited because most of the participants where related to autism organizations or specialized supporting services. We were not able to obtain enough information from autistic people who were not related to that network, so it is not clear whether our results can be generalized to the whole ASD population in Spain, even being coincident, in some extent, with those obtained in similar studies in Europe (3, 58).

This sampling bias may be especially relevant for women and older participants. As already mentioned, we have observed from our registry that the socio-economic, educational and health context of women applicants is usually more disadvantageous compared with male counterparts and globally, adults (especially elderly persons) with ASD are underrepresented in research, and so they are in the present study.

Finally, comparisons with the non-ASD population could not be made, and for some analyses, such as the distribution of genetic disorders, our sample size was too small to allow making any inferences.

#### 4.4.2. Future research

According to our findings and the related scientific literature, it is urgent to enhance the autistic community access to health, improving prevention, identification and management of the conditions that affect their physical and emotional wellbeing. This should include up-to-date training for health professionals regarding ASD, as well as providing resources and guidance to prevent, diagnose and treat comorbidities in a timely and effective manner, including more routine health check-ups and promoting healthy lifestyle changes.

In the future, it will be as well necessary to explore how the aforementioned and other co-occurring conditions impact autistic people’s QoL, their emotional well-being and adaptive functioning, and are subject to sex-based differences.

To increase sample representativeness, further epidemiologic studies with large cohorts are also needed, especially with elder adults and those who are not related to the ASD specialized organizations or do not usually receive any support services. There is a lack of scientific information on the health profile of people with ASD who do not have access to those networks, probably because they have less information and opportunities to participate in health research too. The specific ASD conditions that favor membership in associations could also be linked to health variables, or maybe these organizations support their members in a way that influences their health needs (73). Data from typical controls such as health records, registries and surveys will also be necessary to compare the prevalence of comorbidities in ASD to the general population and pinpoint any associated risk factors.

From a public health perspective, other risk factors such as poor nutritional habits, little physical activity, long-term psychopharmacological treatments and institutionalization increase health problems morbidity among autistic people (28). It has also been suggested that children, adolescents and young adults on the spectrum are more vulnerable to health comorbidities than non-autistic population (22, 24, 25, 74). Finally, difficulties in accessing health care services have also been described for people with ASD, translating into diagnostic and treatment delays (26, 28, 29). All of these areas are amenable to further investigation.

Although the conclusions are still speculative, there seems to exist an association with maternal lifestyle and subsequent diseases (obesity, diabetes, epilepsy and antiepileptic drugs, among others), exposure to specific nutrients and pollutants during pregnancy, advanced parental age and birth complications associated with neonatal hypoxia/ischemia (56, 75–77). Unfortunately, those risk conditions were not included in the registry, and more research is needed to explore their contribution to ASD etiology.

Even though autistic people utilize more health care resources (outpatient visits, emergency room services and hospitalization), they are also more likely to report unmet medical needs, low satisfaction regarding the medical attention received and poor inter-personal communication with health care providers (29). There is an urgent need to increase research that captures the autistic community perspective about those challenges and barriers.

Qualitative and quantitative mixed methods should be applied to identify the key factors that affect the health status and wellbeing of autistic people, taking advantage of the strengths of both approaches and enriching the research results (78, 79).

Also, improving participatory research is necessary, where people on the autism spectrum have an active role in prioritizing the research objectives and how to reach them, and provide feedback to be subsequently analysed and interpreted (80, 81).

To contribute to these efforts, our study has described the health challenges that require urgent awareness and at least some of the main factors (age, sex, and ID) that increase the vulnerability of people on the autism spectrum and deteriorate their wellbeing and QoL. There is a need to determine what factors besides ID contribute to shaping adaptive functioning in autism and QoL, for instance, mental and physical health status, social and workplace inclusion, access to educational, social participation, specialized support resources, and equality of opportunities. The extent to which global health status influences all these outcomes, especially in adulthood and middle age and beyond, remains to be clarified, and it is one of the main priorities considered in the public policies related to ASD around the world (3, 62).

## Data availability statement

The raw data supporting the conclusions of this article will be made available by the authors, without undue reservation.

## Ethics statement

The studies involving human participants were reviewed and approved by Ethics Committee of the Universidad Autónoma de Madrid (CEI-105-2039). Written informed consent to participate in this study was provided by the participants or their legal guardian/next of kin.

## Author contributions

RV-F, MP-S, and CH-L contributed to the study conception and design, and were involved in the material preparation, acquisition, analysis, and interpretation of the data. RV-F and MP-S involved in the drafting of the manuscript. GB-R involved in the analysis and interpretation of the data. CH-L, MV-C, GB-R, and FC-M made substantial contributions to the final draft of the manuscript. All authors read and approved the final manuscript for its publication.
