# Supplementary material for: Characterizing the physical and mental health profile of children, adolescents and adults with autism spectrum disorder in Spain
Source: Front Psychiatry. 2023 Mar 8;14:1088727. doi: 10.3389/fpsyt.2023.1088727 (PMC10031816; doi:10.3389/fpsyt.2023.1088727)
Supplement: Supplementary file 1 [file Data_Sheet_1.PDF]

CONFEDERACIÓN AUTISMO ESPAÑA

ENTIDAD ___________________________________

ID. Iniciales del Nombre y Apellidos_______________

ID. DNI_________________________

******* VARIABLES SOCIALES Y ANTECEDENTES FAMILIARES*******

**P2. Sexo**	
Hombre	
Mujer	

**P3. Fecha de nacimiento**
_ _/_ _ / _ _ _ _

**P4. ¿En qué provincia reside habitualmente?**			
Albacete		León	
Alicante		Lleida	
Almería		Lugo	
Álava		Madrid	
Asturias		Málaga	
Ávila		Murcia	
Badajoz		Navarra	
Baleares, Islas		Ourense	
Barcelona		Palma, las	
Bizkaia		Pontevedra	
Burgos		Rioja, La	
Cáceres		Salamanca	
Cádiz		Santa Cruz de Tenerife	
Cantabria		Segovia	
Castellón		Sevilla	
Ciudad Real		Soria	
Córdoba		Tarragona	
Coruña		Teruel	
Cuenca		Toledo	
Guipúzcoa		Valencia	
Girona		Valladolid	
Granada		Zamora	
Guadalajara		Zaragoza	
Huelva		Ceuta	
Huesca		Melilla	
Jaén			

**P4.2. Localidad**	

**P5. Entidad a la que se encuentra vinculado/a**	
- **Otra Entidad 2**	
- **Otra Entidad 3**	

**P6. Nacimiento pretérmino**	
Sí	
No	

→ **Edad en semanas** [ ]

Pasar a P7

**P7. Bajo peso al nacimiento**	
Sí	
No	

→ **¿Cuántos Kg?** [ ]

Pasar a P8

**P8. ¿Existen antecedentes familiares con TEA?**	
Sí	
No	

→ **¿Quién/es?** [ ]

Pasar a P9

******* DIAGNÓSTICO CLÍNICO*******

**P9. Diagnóstico clínico** (Código CIE-10)	
Diagnóstico desconocido o no confirmado mediante informe clínico o educativo	
Diagnóstico o clasificación criterios DSM 5. TEA con déficit intelectual acompañante (F84.0 + F70.9)	
Diagnóstico o clasificación criterios DSM 5. TEA sin déficit intelectual acompañante (F84.0)	
Diagnóstico o clasificación criterios DSM 5.<br>Asociado a una afección médica o genética, o a un factor ambiental conocido (F84.0 + ___._)	
Diagnóstico o clasificación criterios DSM 5.<br>Asociado a otro trastorno del desarrollo neurológico, mental o del comportamiento (F84.0 + F70._)	
Diagnóstico o clasificación criterios DSM 5. Con catatonIa. (F84.0 + F06.1)	
Diagnóstico- Clasificación criterios DSM-IV. Trastorno autista (F84.0)	
Diagnóstico- Clasificación criterios DSM-IV. Síndrome de Asperger (F84.5)	
Diagnóstico- Clasificación criterios DSM-IV. Trastorno Generalizado Desarrollo- No Especific. (F84.9)	
Diagnóstico- Clasificación criterios DSM-IV. Síndrome de Rett (F84.2)	
Diagnóstico- Clasificación criterios DSM-IV. Trastorno desintegrativo infantil (F84.3)	
Otro ¿Cuál?	

**P9.1. Fecha del diagnóstico TEA confirmado**	_ _ /_ _ / _ _ _ _

**P9.2. ¿Dónde se ha realizado?**	
Ámbito sanitario publico	
Ámbito sanitario privado	
Ámbito educativo público	
Ámbito educativo privado	
Entidad o servicio especializado en TEA. Centro privado	
Entidad específica en TEA (asociación, fundación…)	
Otro tipo de servicio ¿Cuál?	

**P9.2.2 ¿En qué Comunidad/Ciudad Autónoma se ha realizado el diagnóstico?**	

**P10. Evaluación de cociente intelectual**	
**Sí**	
**No**	

→ **Puntuación** (P.10.1) [ ]

Pasar a P11

**P10.2 ¿Tiene discapacidad intelectual?**	
**Sí**	
**No**	

**P10.3 Fecha de realización del mismo**	_ _ /_ _ / _ _ _ _

**P10.4 Prueba utilizada**	
WPPSI-IV	
WPPSI-III	
WISC-IV	
WISC-R	
WAIS-IV	
Otras ¿Cuál?	

**Prueba utilizada**	**Comprensión verbal**	**Visoespacial**	**Razonamiento fluido**	**Memoria de trabajo**	**Velocidad de procesamiento**
**WPPSI-IV**					
**CI Total**					

**Prueba utilizada**	**Verbal**	**Manipulativa**	**Velocidad de procesamiento**
**WPPSI-III**			
**CI Total**			

**Prueba utilizada**	**Comprensión verbal**	**Razonamiento perceptivo**	**Memoria de trabajo**	**Velocidad de procesamiento**
**WISC-IV**				
**CI Total**				

**Prueba utilizada**	**CI Verbal**	**CI Manipulativa**
**WISC-R**		
**CI Total**		

Prueba utilizada	Comprensión verbal	Razonamiento perceptivo	Memoria de trabajo	Velocidad de procesamiento
WAIS-IV				
CI Total				

Prueba utilizada				
OTRA				
CI Total				

******* SALUD Y TRASTORNOS COMÓRBIDOS*******

P11. Trastornos genéticos asociados	
Sí	
No	

Sí → **¿Cuál/es?** ____________

No → **Pasar a P12**

P12. Alteraciones diagnosticadas del sistema nervioso	
Epilepsia y crisis epilépticas recurrentes (G40.90_)	
Espina bífida u otras malformaciones congénitas del sistema nervioso (Q05)	
Parálisis cerebral (G80)	
SAF (Síndrome alcoholismo fetal) (P04.3)	
Otras ¿cuáles?	

P13. Trastornos comórbidos clínicamente diagnosticados	
Trastornos depresivos (F32)	
Trastornos de ansiedad (F41)	
Trastorno obsesivo compulsivo (F42.8, F42.9)	
Trastorno de la alimentación (F50)	
Trastornos de control de los impulsos y conductas disruptivas (F63)	
Otros, ¿cuál?	

P14. Enfermedades crónicas diagnosticadas (diagnóstico médico confirmado)	
Trastornos de la glándula tiroides (E00-E07)	
Diabetes (E10-E14)	
Sobrepeso u obesidad (E66)	
Alteraciones metabólicas (E70-E90)	
Trastornos del sueño (F51)	
Úlcera gástrica, gastritis o esofagitis (K25, K29, K20)	
Trastornos intestinales (K59)	
Dermatitis y eccema (L30)	
Urticaria (L50)	
Anomalías dentofaciales y otros trastornos mandibulares (M26)	
Enfermedades del riñón y otros órganos del aparato urinario (N10-N39)	
Trastornos de próstata u otros órganos genitales masculinos (N40-N51)	
Trastornos del tracto genital femenino (N80-N98)	
Otros trastornos crónicos de salud. ¿Cuál/es?	

**P15. Recibe tratamiento psicofarmacológico en la actualidad**	**Sí**	**No**	**¿Cuál/es?**
Antipsicóticos			
Metilfenidato			
Ansiolíticos			
Anticonvulsivantes-antiepilépticos			
Antidepresivos			
Tratamientos hormonales			
Melatonina			
Otros. ¿Cuál/es?			

******* VALORACIÓN DE DISCAPACIDAD, DEPENDENCIA Y PRESTACIONES ASOCIADAS*******

**P16. ¿Tiene certificado de discapacidad?**		
Sí		**Pasar a P16.2**
No		**Pasar a P17**

**P16.2 ¿Qué tipo de certificado?** (Contestar solo si ha respondido Sí en la P16)	
Provisional	
Definitivo	

**P 16.3 Calificación reconocida**	
**P16.4 Fecha de dictamen actual**	_ _ /_ _ / _ _ _ _
**P16.5 Ciudad de expedición**	

**P16.6 Reconocimiento positivo en Baremo, dificultades de movilidad para utilizar transportes colectivos**		
Sí		
No		**Pasar a P17**

**Factores recogidos en el Baremo** (Contestar solo si ha respondido Sí en la P16.6)	
Confinado en silla de ruedas	
Depende absolutamente de dos bastones para deambular	
Puede deambular, pero presenta conductas agresivas o molestas de difícil control, a causa de graves deficiencias intelectuales que dificultan la utilización de medios normalizados de transporte.	

P17. ¿Valoración del grado de dependencia?	
Sí	
No	

Pasar a P17.1

Pasar a P19

P17.1 Grado de dependencia	
Grado I- Dependencia moderada	
Grado II- Dependencia severa	
Grado III- Gran dependencia	

P18. Percibe prestaciones o servicios, vinculados al reconocimiento de la situación de Dependencia	
Sí	
No	

Pasar a P18.1

Pasar a P19

P18.1 ¿Qué tipo de prestaciones o servicios percibe?	
Económica vinculada al servicio (Especificar servicio)	
Económica para cuidados no profesionales en el entorno familiar y apoyo a cuidadores no profesionales	
Económica de asistencia personal	
Servicio de Prevención de la situación de dependencia	
Servicio de Teleasistencia	
Servicio de Ayuda al Domicilio	
Servicio de Centro de Día y de Noche	
Servicio de Atención Residencial	

P19. Incapacidad Laboral. Solo mayores de 18 años	
Sí	
No	

P19.1 ¿Qué tipo?	
Temporal	
Permanente parcial para la profesión habitual	
Permanente total para la profesión habitual	
Permanente absoluta para todo trabajo	
Permanente gran invalidez	

P20. Modificación de la capacidad (Incapacitación judicial)	
Sí	
No	

Pasar a P20.1

Pasar a P21

P20.1				
**Tipo de incapacitación**	Total		Parcial	
**Figura de guarda**	Tutor/a		Curador/a	

P21. ¿Recibe algún tipo de prestación o beca?		
Sí		**Pasar a P21.1**
No		**Pasar a P22**

P21. 1 ¿Qué tipo de prestación o beca?	
Pensión no contributiva de invalidez	
Pensión de orfandad	
Subsidio por movilidad y compensación por gastos de transporte SMGT	
Prestación social de asistencia sanitaria y prestación farmacéutica ASPF	
Subsidio de garantía de ingresos mínimos	
Prestación por hijo/a, a cargo	
Subsidio por ayuda de tercera persona (SATP)	
Beca del Ministerio de Educación	
Otras becas públicas. ¿Cuál?	
Becas privadas. ¿Cual?	
Otras prestaciones económicas ¿Cuál?	

P22. Entorno en el que reside la persona	
Hogar familiar	
Vivienda específica para personas con TEA	
Vivienda general para personas con discapacidad intelectual o del desarrollo	
Piso tutelado con apoyos continuados	
Vivienda independiente con apoyos puntuales. Solo/a	
Vivienda independiente con apoyos puntuales. Con pareja	
Vivienda independiente con apoyos puntuales. Con pareja e hijos	
Vivienda independiente con apoyos puntuales. Con amigos/as, compañeros/as…	
Vivienda independiente sin apoyo. Solo/a	
Vivienda independiente sin apoyo. Con pareja	
Vivienda independiente sin apoyo. Con pareja e hijos/as	
Vivienda independiente sin apoyo. Con amigos/as, compañeros/as…	
Estancia combinada (residencia en vivienda asociada a una entidad y en hogar familiar	
Otros. ¿Cuál/es?	

******* EDUCACIÓN*******

P23. Nivel de estudios finalizado	
No procede, menor de 10 años	
Educación Básica Obligatoria (EBO)	
Estudios primarios primer ciclo (1º y 2º)	
Estudios primarios	
Programa de transición a la vida adulta	
Educación secundaria Obligatoria- primer ciclo (1º y 2ª)	
Educación secundaria Obligatoria	
Bachillerato	
Formación profesional básica	
Ciclo Formativo Grado Medio	
Ciclo Formativo Grado Superior	
Estudios universitarios (grado, diplomatura o licenciatura)	
Estudios de posgrado (doctorado, máster o equivalente)	

P24. En qué modalidad se encuentra escolarizado/a en la actualidad		
Educación especial		**Pasar a P24.1**
Educación ordinaria		**Pasar a P24.2**

P24.1 EDUCACIÓN ESPECIAL

Tipo de centro en el que se encuentra escolarizado	
Centro de Educación Especial específico para alumnado con TEA	
Centro de Educación Especial para alumnado con discapacidad	
Unidad de educación especial en centro educativo ordinario	
Escolarización combinada centro ordinario-específico	

Etapa en la que se encuentra escolarizado/a:	
Educación Infantil Especial (3-5 años)	
Enseñanza Básica Obligatoria (6-16/18 años)	
Programas de Transición a la Vida Adulta (17/19-21 años)	

P24.2 EDUCACIÓN ORDINARIA

Tipo de centro en el que se encuentra escolarizado	
Centro de Educación Ordinaria	
Centro de Educación Ordinaria de escolarización preferente para alumnado con TEA	
Centro de educación ordinaria con apoyos a alumnado con necesidades específicas de apoyo educativo	
Escolarización combinada centro ordinario-específico	

**Etapa en la que se encuentra escolarizado/a:**	
Educación Primaria – Primer ciclo	
Educación Primaria – Segundo ciclo	
Educación Secundaria Obligatoria- Primer ciclo	
Educación Secundaria Obligatoria- Segundo ciclo	
Bachillerato	
Ciclo formativo de formación profesional básica	
Ciclos formativos grado medio de formación profesional específica o equivalente	
Bachillerato	
Formación Profesional Básica	
Ciclo Formativo de Grado Medio	
Ciclo Formativo de Grado Superior	
Enseñanzas universitarias- Grado o Licenciatura	
Enseñanzas universitarias-Diplomatura	
Enseñanzas universitarias- Master	
Enseñanza universitarias- Doctorado	
Educación no reglada	
Ninguna de las anteriores ¿Cuál?	

******* EMPLEO/OCUPACIÓN PRINCIPAL*******

CONTESTAR SOLO LOS/AS MAYORES DE 16 AÑOS

**P25. Actividad económica u ocupación**	
Empleado/a por cuenta ajena (con contrato laboral) **pasar a P27**	
Empleado/a por cuenta propia (con alta en régimen de trabajador autónomo) **pasar a P28**	
Desempleado/a, inscrito/a como demandante de empleo **pasar a P26**	
Desempleado/a, inscrito/a como demandante de empleo realizando prácticas laborales **pasar a P26**	
Desempleado/a, inscrito/a como demandante de empleo realizando cursos de formación complementaria **pasar a P26**	
Desempleado/a, NO inscrito/a como demandante de empleo **pasar a P26**	
Desempleado/a, NO inscrito/a como demandante de empleo realizando prácticas laborales **pasar a P26**	
Desempleado/a, NO inscrito/a como demandante de empleo realizando cursos de formación complementaria	
Estudiante **pasar a P31.1**	
Jubilado/a **pasar a P31.1**	
Otra situación. ¿Cuál?	

P26. Si la persona se encuentra en situación de desempleo ¿dónde mantiene su ocupación principal?	
En casa	
Cetro de día específico para personas con TEA	
Centro de día generalista para personas con discapacidad	
Centro ocupacional	
Otros servicios de apoyo. ¿Cuáles?	

P27. Si la persona trabaja por cuenta ajena, ¿dónde desarrolla su actividad laboral?	
Centro especial de empleo/enclave laboral	
Empresa ordinaria con apoyos (empleo con apoyo)	
Empresa ordinaria sin apoyo	
Administración pública con apoyo	
Administración pública sin apoyo	

P28. Si la persona trabaja por cuenta propia, ¿Cuál es la forma jurídica de su negocio?	
Sociedad (Limitada, Anónima…)	
Autónomo	
Cooperativa	
La actividad no está dada de alta	
Otro ¿Cuál?	

Contestar solo aquellas personas que trabajan

**P29. Tipo de jornada** (Contestar solo aquellas personas que tienen un empleo)	
Jornada total	
Jornada parcial	

P30. Número de horas que trabaja a la semana (establecidas por contrato)	

********* INTERVENCIONES/TERAPIAS RECIBIDAS A LO LARGO DEL ÚLTIMO AÑO*********

P31.1 Programas de intervención conductual	
EIBI (Intervención conductual temprana intensiva)	
ABA (Análisis aplicado de la conducta)	
LEAP (Learning Experiences and Alternative Program for Preschoolers and their parents)	
ESDM (Early Start Denver Model)	
Apoyo Conductual Positivo	
Otras. Especificar cuál/es	

P31.2 Intervenciones basadas en el desarrollo	
Floortime	
RDI (Relationship Development Intervention)	
SCERTS	
Otras. Especificar cuál/es	

**P32. 3 Intervenciones centradas en la comunicación y el lenguaje**	
Sistemas alternativos/ aumentativos de comunicación- Apoyos visuales (objetos reales, pictogramas o escritura)	
Sistemas alternativos/ aumentativos de comunicación -Programa de habla signada (comunicación total) de Benson Schaeffer	
PECS (Sistema de Comunicación por Intercambio de Imágenes)	
Apoyos visuales en el entorno para favorecer la comprensión (horarios, calendarios, instrucciones, etc.)	
Otras. Especificar cuál/es	

**P31.4 Programas para el fomento de las competencias de comunicación social y de interacción**	
Historias y guiones sociales	
Habilidades Sociales en grupo	
RDI (Intervención para el desarrollo de las relaciones)	
Otras. Especificar cuál/es	

**P31.5 Adaptaciones en el contexto/entorno**	
Enseñanza estructurada- TEACCH	
Otras. Especificar cuál/es	

**P31.6 Intervenciones centradas en área sensoriomotriz**	
Terapia en integración sensorial	
Otras. Especificar cuál/es	

**P31.7 Intervención clínica (psicológica)**	
Intervención cognitivo-conductual	
Otras. Especificar cuál/es	

**P31.8 Intervenciones nutricionales**	
Dietas libres de gluten y caseína	
Suplementos de ácidos Omega 3	
Suplementos vitamínicos	
Otras. Especificar cuál/es	

**P31.9 Otras intervenciones**	
Terapia asistida con animales	
Musicoterapia	
Homeopatía	
Otras. Especificar cuál/es	

******* APOYOS Y SERVICIOS RECIBIDOS DESDE LA ENTIDAD *******

**P32. Información Orientación y Asesoramiento**	

**P.33 Conciliación de la vida familiar y/o laboral**	
Programas de primero de la mañana o últimos de la tarde	
Programas de fin de semana	
Campamentos urbanos / Centros abiertos	
Respiro familiar (cualquier día de la semana)	
Actividades extraescolares	
Acompañamiento a la persona con TEA en actividades puntuales	
Apoyo en situaciones de emergencia	
Apoyo en el domicilio	
Otro. Por favor especifique.	

**P34. Asistencia personal**	

**P.35 Apoyo psicológico y emocional**	
Apoyo psicológico especializado para personas con TEA	
Otro. Por favor especifique.	

**P.36 Diagnóstico y evaluación**	
Diagnóstico clínico y evaluación	
Seguimiento	
Otro. Por favor especifique.	

**P.37 Intervención especializada y/o apoyo**	**Sí**	**No**	**¿En contexto natural?** Señalar en caso afirmativo
Atención temprana de 0 a 3 años			
Atención temprana de 3 a 6 años			
Intervención/terapia individual: lenguaje, habilidades sociales, etc.			
Otro. Por favor especifique.			

**P.38 Ocio y tiempo libre/educación física**	
Actividades de ocio en instalaciones de la entidad	
Actividades de ocio en entornos comunitarios	
Actividades vacacionales (campamentos, escuela de verano...)	
Actividades físicas y deportivas	
Actividades artísticas	
Otro. Por favor especifique.	

**P.39 Apoyo especializado para la promoción de la salud**	
Desensibilización para revisiones médicas (dentista, atención primaria, etc...)	
Acompañamiento a revisiones médicas	
Apoyo en hospitalización	
Seguimiento nutricional	
Seguimiento neuropsiquíatrico, farmacológico,...	
Fisioterapia	
Educación sexual	
Otro. Por favor especifique.	

**P.40 Educación y servicios de apoyo educativo**	
Servicios de educación especial específicos para alumnado TEA: Infantil (3-6 años)	
Servicios de educación especial específicos para alumnado con TEA: Básica obligatoria especial (6-21 años)	
Servicios de educación especial específicos para alumnado con TEA: Programas de transición a la vida adulta	
Servicios de apoyo a la educación ordinaria- Educación infantil	
Servicios de apoyo a la educación ordinaria- Educación Primaria	
Servicios de apoyo a la educación ordinaria- Educación Secundaria	
Servicios de apoyo a la educación ordinaria- Bachillerato	
Servicios de apoyo a la educación ordinaria- Universidad	
Otro. Por favor especifique.	

**P.41 Empleo**	
Formación prelaboral o formación en prácticas laborales	
Programas de empleo con apoyo	
Orientación laboral	
Enclave laboral	
Otro. Por favor especifique	

**P.42 Atención diurna para personas adultas**	
Unidades de día/ centros de día	
Unidades integradas en la comunidad	
Otro. Por favor especifique	

**P43. Centro ocupacional**	

**P.44 Vivienda**	
Vivienda/residencia para personas con grandes necesidades de apoyo	
Vivienda/piso tutelado	
Vivienda de estancia temporal	
Servicio de respiro con pernocta	
Otro. Por favor especifique.	

**P.45 Apoyos al envejecimiento activo**	
Servicio/programa de apoyo en la vejez	
Otro. Por favor especifique	
